# Environmental noise stress disturbs commensal microbiota homeostasis and induces oxi-inflammmation and AD-like neuropathology through epithelial barrier disruption in the EOAD mouse model

**DOI:** 10.1186/s12974-020-02053-3

**Published:** 2021-01-06

**Authors:** Huimin Chi, Wa Cao, Ming Zhang, Donghong Su, Honglian Yang, Zhe Li, Chao Li, Xiaojun She, Kun Wang, Xiujie Gao, Kefeng Ma, Pengfang Zheng, Xiaofang Li, Bo Cui

**Affiliations:** 1 Institute of Environmental and Operational Medicine, Chinese Academy of Military Medical sciences, Tianjin, China; 2 School of Public Health and Management, Weifang Medical University, Weifang, China; 3 College of Public Health, North China University of Science and Technology, Tangshan, China; 4 Shenzhen Prevention and Treatment Center for Occupational Diseases, Shenzhen, China; 5 Zibo Center for Disease Control and Prevention, Zibo, China; 6 School of Public Health and Management, Binzhou Medical University, Yantai, China

**Keywords:** Environmental noise, Microbiome–gut–brain axis, Oxidative stress, Inflammation, Early-onset Alzheimer’s disease (EOAD), APP/PS1 mouse

## Abstract

**Background:**

Both genetic factors and environmental hazards, including environmental noise stress, have been associated with gut microbiome that exacerbates Alzheimer’s disease (AD) pathology. However, the role and mechanism of environmental risk factors in early-onset AD (EOAD) pathogenesis remain unclear.

**Methods:**

The molecular pathways underlying EOAD pathophysiology following environmental noise exposure were evaluated using C57BL/6 wild-type (WT) and APP/PS1 Tg mouse models. The composition differences in intestinal microbiota were analyzed by 16S rRNA sequencing and Tax4Fun to predict the metagenome content from sequencing results. An assessment of the flora dysbiosis-triggered dyshomeostasis of oxi-inflamm-barrier and the effects of the CNS end of the gut–brain axis was conducted to explore the underlying pathological mechanisms.

**Results:**

Both WT and APP/PS1 mice showed a statistically significant relationship between environmental noise and the taxonomic composition of the corresponding gut microbiome. Bacterial-encoded functional categories in noise-exposed WT and APP/PS1 mice included phospholipid and galactose metabolism, oxidative stress, and cell senescence. These alterations corresponded with imbalanced intestinal oxidation and anti-oxidation systems and low-grade systemic inflammation following noise exposure. Mechanistically, axis-series experiments demonstrated that following noise exposure, intestinal and hippocampal tight junction protein levels reduced, whereas serum levels of inflammatory mediator were elevated. Regarding APP/PS1 overexpression, noise-induced abnormalities in the gut–brain axis may contribute to aggravation of neuropathology in the presymptomatic stage of EOAD mice model.

**Conclusion:**

Our results demonstrate that noise exposure has deleterious effects on the homeostasis of oxi-inflamm-barrier in the microbiome–gut–brain axis. Therefore, at least in a genetic context, chronic noise may aggravate the progression of EOAD.

## Background

Early-onset Alzheimer’s disease (EOAD) is a rare form of neurodegeneration disease with a large genetic basis that is only partially understood. Only a limited proportion of EOAD cases are linked to autosomal dominant mutations in the amyloid precursor protein (APP), presenilin 1 (PSEN1), or presenilin 2 (PSEN2) genes [1]. One possible explanation is that environmental factors may promote AD pathogenesis. For example, early-life stress causes a neuroinflammatory response in a transgenic mouse model of EOAD and may aggravate the neuropathology and alter disease progression [2]. Environmental noise is one of the most pervasive health hazards [3–5], which has also been associated with AD-like cognitive dysfunction as well as persistent tau and amyloid-β (Aβ) pathology in murine model [6–10]. Furthermore, environmental noise alone or in combination with ApoE4 or aging, important internal risk factors for sporadic late-onset AD, can aggravate the occurrence and development of AD [11, 12]. Environmental stress may activate the APP/PS1 gene, which may further cause cognitive deficits and an impaired neuroinflammatory response to amyloid pathology [13]. This indicates that the combined effects of environmental noise and genetic risk factors may have a greater impact on EOAD progression than exposure to individual factors alone.

Commensal gut microbiota dysbiosis closely related to environmental hazards [14] may have both causal and consequent links to oxidative stress, underlying several neurodegenerative disorders, including AD [15]. We have previously demonstrated that noise stress negatively affects the composition and diversity of the gut microbiota and impairs barrier functions in the intestine and hippocampus [16]. Accordingly, we hypothesized that the dysbiosis of gut flora caused by internal and external environmental hazards would induce intestinal oxidative stress, which would subsequently destroy the epithelial barrier function of the gut–brain axis and promote the AD-like pathology.

The present study aimed to evaluate the gut and neuro-molecular pathways involved in EOAD pathophysiology following environmental stress. Based on our previous results, we particularly focused on chronic noise exposure and APP/PS1 overexpression, both of which can trigger the onset of EOAD pathology in a presymptomatic stage of the disease at 3 months of age in a APP/PS1 mouse model [17]. Moreover, we explored early biochemical and molecular pathological clues that might permit better understanding of the link between EOAD and environmental risks.

## Methods

### Animals and experimental groups

Fifty-three 60-day-old male B6C3-Tg (APPswe, PSEN1dE9)/NJU (APP/PS1) and C57BL/6Nju wild-type (WT) mice were purchased from Nanjing Biomedical Research Institute and used for this study. To avoid the cage effect of co-housing animals from microbiome transfer [18], each mouse was housed in individually ventilated cages under specific-pathogen-free (SPF) systems, with a 12-h light/12-h dark cycle (lighting phase from 06:00 to 18:00 daily) in a temperature-controlled room maintained at 23 ± 2 °C and a relative humidity of 45–55%. Mice had free access to food (Laboratory animals―Nutrients for formula feeds, GB 14924.3-2010) and water in their cages and were habituated to the conditions for 5 days prior to the experiment procedure.

All APP/PS1 and WT mice were randomly divided into four groups (*n* = 10–15 in a group), with a 2 × 2 factor design (independent factors: exposure and genotype). The noise (WT mice) and noise+APP/PS1 (APP/PS1 mice) groups were exposed to white noise at 98 dB SPL (4 h/day for 30 days from 8:00 to 12:00). The control mice (WT mice) and APP/PS1 mice were placed in another room with background noise under 40 dB SPL. After 30 consecutive days of exposure, all animals in this study were sacrificed. Hippocampus tissue, blood, cecal tissue, and their contents were collected immediately for biochemical analyses and stored at − 80 °C until use. All animal experimental protocols were approved by Tianjin Institute of Environmental and Operational Medicine Animal Use and Research Committee.

### Noise exposure set-up

The noise exposure procedure followed the method reported in our previous work [19]. Briefly, white noise was generated using a sound signal generator (BK 3560C, B&K Instruments, Denmark), amplified with a power amplifier, and delivered through a loudspeaker. The frequency spectrum of the sound signal from the generator was in the range of 20–20 k Hz. Mice were placed in wire-mesh cages and exposed to white noise in a reverberation chamber at the center of the sound field, where the loudspeaker was suspended directly above the cages.

### Sequencing of the 16S rRNA gene in microbiota

We performed amplicon sequencing of 16S rRNA as in our previous research [16]. Briefly, DNA was extracted using the cetyltrimethylammonium bromide/sodium dodecyl sulfate method from cecal contents. Distinct regions of 16S rRNA genes were amplified with specific primers (e.g., 16S V4: 515F-806R, 18S V4: 528F-706R, and 18S V9: 1380F-1510R) with the barcode. PCR reactions were performed with Phusion® High-Fidelity PCR Master Mix (New England Biolabs) and were analyzed to evaluate bacterial diversity using Illumina Hiseq (Novogene Bioinformatics Technology, Beijing, China). Sequences were analyzed using the Quantitative Insights Into Microbial Ecology software (http://qiime.org/). The most abundant sequence of each operational taxonomic unit (OTU) was selected as a typical sequence, of which the taxonomic information was annotated and classified by applying the Ribosomal Database Project classifier.

### Taxonomic and functional analysis

The ɑ diversity data of the samples were obtained by the mothur algorithm, β diversity was analyzed by UniFrac distance, and LEfSe (linear discriminant analysis coupled with effect size measurements) and Metastat analysis (a tool for meta-analysis) were used to identify microbial gene characteristics or microbial-related functional pathways among groups. Linear discriminant analysis (LDA) was used to evaluate the statistical efficiency of biomarkers. To further understand the specific functions of each group of bacteria, 16S high-throughput sequencing data were used to classify the OTUs through the SILVAngs platform based on the SILVA database. The 16S copy number was then standardized according to the National Center of Biotechnology Information genome annotation. Finally, the prediction of the microbial community function was realized by constructing a linear relationship between the SILVA classification and pronuclear classification in the KEGG database.

### Analyses for T-AOC, GST, CAT, GSH-Px, and SDH activity

Total antioxidant capacity (T-AOC, No. A015-2-1, ABTS method) and activities of catalase (CAT, No. A007-1-1, Visible light method), glutathione S-transferase (GST, No. A004-1-1, Colorimetric method), and glutathione peroxidase (GSH-Px, No. A005-1-2, Colorimetric method) in the cecal contents and succinate dehydrogenase (SDH, No.A022-1-1, colorimetric method) in the colon tissue were analyzed using commercial assay kits (Jiancheng Institute of Biotechnology, Nanjing, China). Detailed operation procedures were performed following the manufacturer’s product manual. The mean value of thrice duplicate samples was presented as the final result for each mouse.

### Determination of gene expression by real-time PCR

Real-time PCR was used to detect the gene expression of oxidation-related enzymes. Total RNA was extracted from the colon tissue using the RNeasy kit and then converted to cDNA via reverse transcription using a transcriptor first strand cDNA synthesis kit according to the manufacturer’s product manual (TaKaRa Bio, Dalian, China). Specific primers and probes designed for mouse *GCLC*, *GSS*, *GSR*, *GSTpi*, *NOX2/4* (NADPH oxidase 2/4), and *GAPDH* were used as described in Table 1, and the *GAPDH* gene was used as an internal reference standard. The essential experimental operation of quantitative real-time PCR followed the procedure of a previous study [11]. Real-time PCR was performed by using gene expression assays-on-demand and a Takara PCR thermal cycler dice real-time system (TaKaRa Bio, Dalian, China). mRNA levels were calculated after normalizing the Ct value to *GAPDH* expression and presented as fold-induction values (2^−ΔΔCt^) relative to those of WT control mice.

**Table 1:** Mouse primer sequences used for quantitative real-time PCR

Gene	Primers
***GCLC***	**F:5′-CTACCACGCAGTCAAGGACC-3′** **R:5′-CCTCCATTCAGTAACAACTGGAC-3′**
***GSS***	**F:5′-AAAGCAGGCCATAGACAGGG-3′** **R:5′-TGAATGGGGCATACGTCACC-3′**
***GSR***	**F:5′-GCGTGAATGTTGGATGTGTACC-3′** **R:5′-GTTGCATAGCCGTGGATAATTTC-3′**
***GSTpi***	**F:5′-ATGCCACCATACACCATTGTC-3′** **R:5′-GGGAGCTGCCCATACAGAC-3′**
***Nox2***	**F: 5′-TGTTTTCATTTCCTCATCAGAAG-3′** **R:5′-CCAACCACACCAGAATGACA-3′**
***Nox4***	**F: 5′-TCTGGAAAACCTTCCTGCTG-3′** **R:5′-CCGGCACATAGGTAAAAGGA-3′**
***GAPDH***	**F:5′-AGGTCGGTGTGAACGGATTTG-3′** **R:5′-TGTAGACCATGTAGTTGAGGTCA-3′**

### Enzyme-linked immunosorbent assay (ELISA)

Colon and hippocampus tissues for ELISA measurements were homogenized in phosphate buffer (Ph = 7.4), the lysates were centrifuged at 3000 rpm for 30 min at 2–8 °C, and the supernatant was collected. The BCA protein quantification assay kit (Thermofisher Scientific) was used to determine the protein concentrations. Levels of ApoA4, interleukin (IL)-6, inducible nitric oxide synthase (iNOS), nuclear factor κB (NF-κB), neutrophil gelatinase-associated lipid delivery protein (NGAL), and NF-E2-related factor 2 (Nrf2) in the intestine; levels of Aβ40 and Aβ42 in the hippocampus; and blood plasma levels of 5-HT, GABA, D-lactose (D-LAC), endotoxin, IL-6, and tumor necrosis factor (TNF) were measured with mouse ELISA kits (BlueGene Biotech, Shanghai, China) strictly according to the manufacturer’s protocol, and a chromogenic substrate (tetramethylbenzidine, TMB) and stop solution were used as blank. The average of duplicate samples was taken as the final result for each mouse.

### Western blot analysis

The frozen colon and hippocampus tissues were homogenized and centrifuged for immunoblot analysis as previously described [16]. Expression of proteins related to epithelial barrier function was immunoblotted using mouse antibodies against phospho-Tau at sites Ser202, Thr205 (AT8, 1:1,000, Invitrogen, USA) and rabbit antibodies against the following proteins: phospho-Tau (Ser404) (1:200, Santa Cruz, USA), claudin 1 (CLDN1,1:1,000, Bioworld, USA), occludin (1:800, Bioworld, USA), tight junction protein 1(ZO-1,1:500, Proteintech, USA), and glyceraldehyde phosphate dehydrogenase (GAPDH, 1:10,000, Proteintech, USA). GADPH served as an internal reference standard for normalization. Peroxidase-conjugated goat anti-rabbit and goat anti-mouse IgG (H+L) (ZSGB-BIO, China) were used as secondary antibody, which was visualized by an enhanced chemiluminescent assay.

### Transmission electron microscopy

After sacrificing two mice from each group, the hippocampus was harvested and fixed with 4% glutaraldehyde in cacodylate buffer. Thin sections (50 nm) were prepared, mounted on copper grids and uranium-lead double stained with saturated aqueous solution of 2% uranium acetate and lead citrate for examination using a TECNAI G 20 TWIN electron microscope (FEI, Hillsboro, OR, USA).

### Statistical analysis

Quantitative data presented in graphs indicate the group mean ± standard deviation and were analyzed by using IBM SPSS Statistics 19.0 and Graphpad Prism 7. Data of REDOX enzyme activity, ELISA, Western blot, and gene expression were analyzed with two-way ANOVA (noise exposure and genotype). The post hoc analyses were performed using Bonferroni tests for multiple comparison when a significant interaction effect was detected. Data of the 16S rRNA gene analysis were assessed by using Wilcoxon rank sum tests. Redundancy analysis was performed using CANOCO 4.5. The differences were considered statistically significant when the *P* value was < 0.05.

## Results

### Chronic noise exposure alters community composition of gut microbiota in APP/PS1 mice

**Fig. 1:**
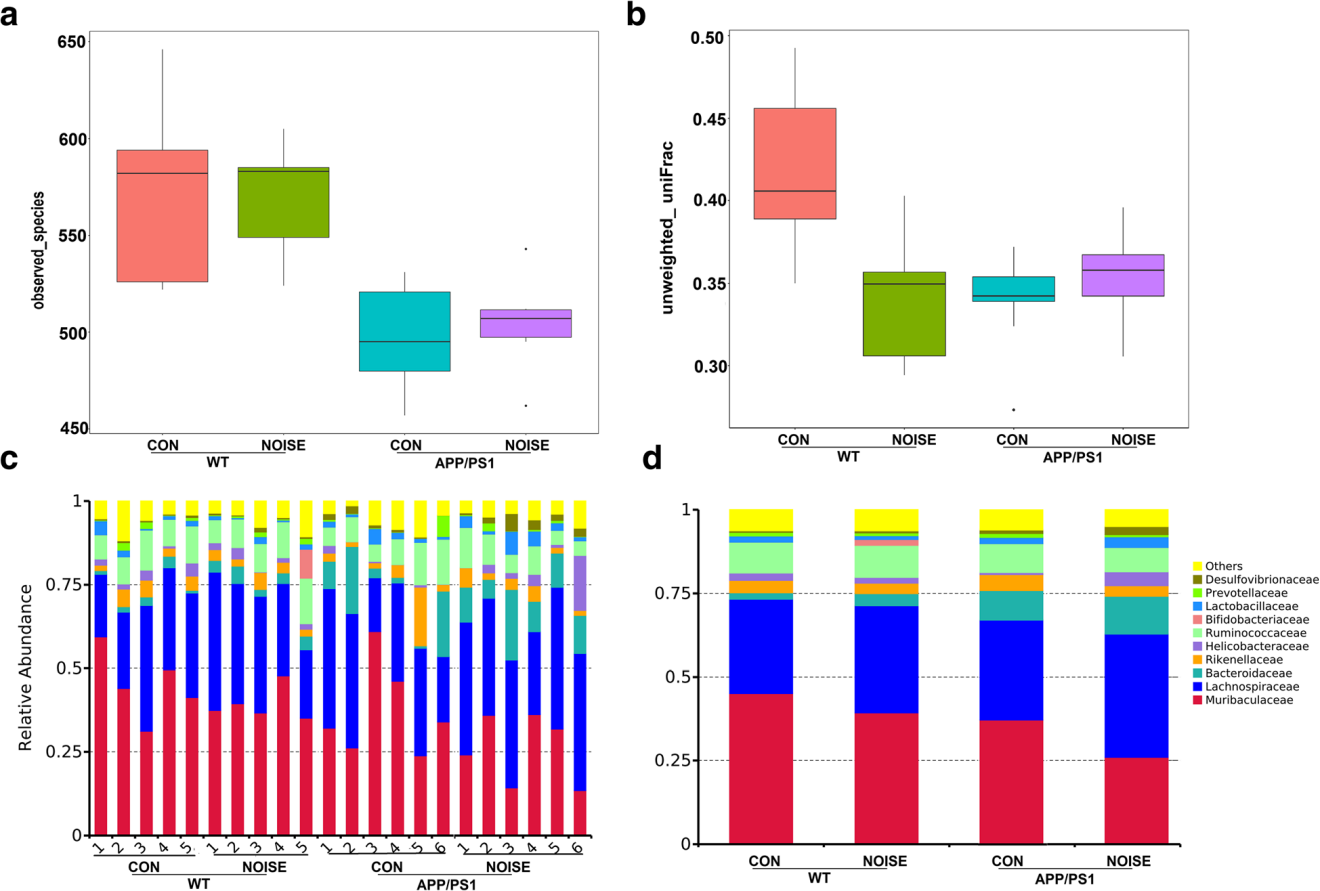
Summary of gut microbial communities in each group. **a** Alpha diversity of the microbial community in each group (*n* = 5–6). **b** Intragroup β-diversity of the microbial community in each group, as measured by unweighted UniFrac distance (*n* = 5–6). **c**, **d** Relative abundances of predominant bacteria at the family level in each sample and group (*n* = 5–6). CON, control groups; NOISE, noise-exposed groups; WT, wild-type mice; APP/PS1, APP/PS1 transgenic mice

We assessed the mouse gut microbiota structure by 16S sequencing-based analysis of gDNA extracted from cecal contents. There were no significant differences in ɑ-diversity or β-diversity (*P* > 0.05) of the microbial community between noise-exposed and control groups in both WT and APP/PS1 mice; however, the microbiota community in noise-exposed or APP/PS1 mice exhibited lesser intragroup diversity than that in WT control mice (Fig. 1a, b). At the family level, *Muribaculaceae*, *Lachnospiraceae*, *Bacteroidaceae*, *Rikenellaceae*, *Helicobacteraceae*, *Ruminococcaceae*, *Bifidobacteriaceae*, *Lactobacillaceae*, *Prevotellaceae*, and *Desulfovibrionaceae* were dominant bacteria taxa (Fig. 1c, d). Notably, taxonomic profiling showed that the relative abundance of *Muribaculaceae* was decreased, while *Bacteroidaceae* increased markedly (Fig. 1d), which demonstrated that the gut microbiota pattern was markedly altered in chronic noise exposure and APP/PS1 mice.

**Fig. 2:**
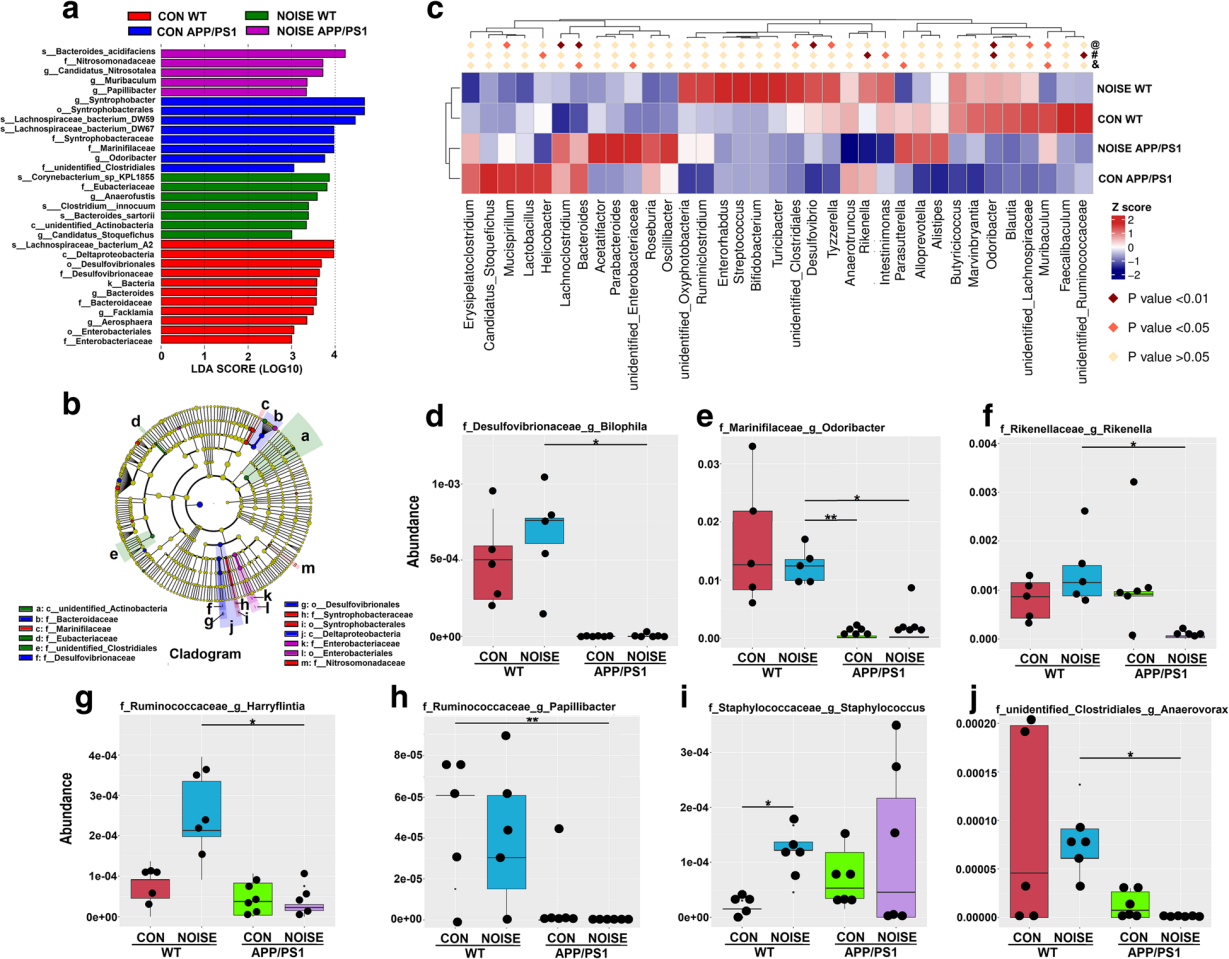
Alterations in gut microbiota composition following chronic noise exposure. **a** The most differentially abundant taxa in each group identified by LDA scores generated from the LEfSe analysis (*n* = 5–6). **b** The enriched taxa in fecal microbiota of mice are represented in cladograms. The central point represents the root of the tree (bacteria), and each ring represents the next lower taxonomic level (phylum to genus). The diameter of each circle represents the relative abundance of the taxon (*n* = 5–6). **c** A heatmap showing Metastat analysis of species with significant differences between groups based on species abundance tables at the bacterial genus level. **d**–**j** Comparison of relative abundance at the bacterial genus level in all groups. *P < 0.05, **P < 0.01, vs. indicated group, Mann–Whitney *U* test. CON, control groups; NOISE, noise-exposed groups; WT, wild-type mice; APP/PS1, APP/PS1 transgenic mice; @, WT NOISE group vs. WT CON group; #, APP/PS1 CON group vs. WT CON group; &, APP/PS1 NOISE group vs WT CON group

LEfSe and Metastat analysis were further used to assess species with significant differences between groups based on species abundance after chronic noise exposure. The structure and predominant bacteria of microbiota in each group were represented as a cladogram, and the greatest difference in taxa from phylum to genus level was identified via the LDA score (Fig. 2a, b). At the genus level, species with significant differences between groups were represented as a heatmap (Fig. 2c). Noise exposure significantly elevated levels of *Staphylococcus* and *Papillibacter* in the WT groups and decreased levels of *Rikenella* and *Anaerovorax* in mouse model of EOAD (Fig. 2d–j).

### Prediction of microbiota functional capacity via Tax4Fun

**Fig. 3:**
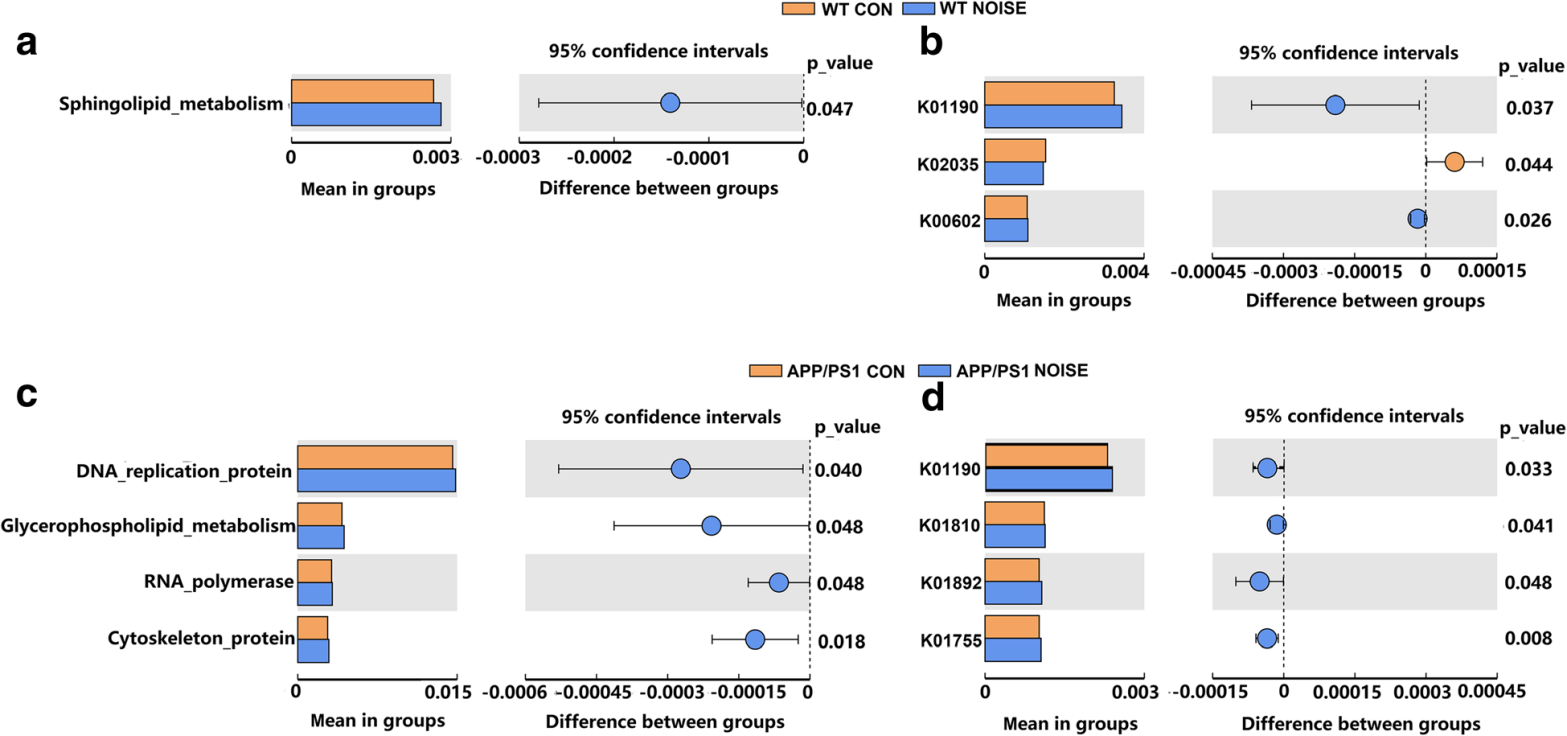
Tax4Fun showing predicted functional capabilities of microbial communities based on 16S rRNA datasets. **a** KOs with significant difference at level 3 between the WT CON group and WT NOISE group. **b** KOs with significant difference at level K between the WT CON group and WT NOISE group. **c** KOs with significant difference at level 3 between the APP/PS1 CON group and APP/PS1 NOISE group. **d** KOs with significant difference at level K between the APP/PS1 CON group and APP/PS1 NOISE group. CON, control groups; NOISE, noise-exposed groups; WT, wild-type mice; APP/PS1, APP/PS1 transgenic mice

We used Tax4Fun to predict the metagenome content and impute the metagenome from our 16S rRNA sequencing results. We found that chronic noise exposure significantly increased KEGG orthologs (KOs) of the sphingolipid metabolism (Level 3 KOs, Fig. 3a) and K01190 and K00602 (Level K, Fig. 3b) in WT mice, while K02035 significantly decreased after noise exposure (Level K, Fig. 3b). Tax4Fun assignment to Level 3 and Level K KOs suggested that the most predicted functional categories in KEGG pathways were significantly altered after noise exposure in APP/PS1 mice, including increased KOs of DNA replication proteins and glycerophospholipid metabolism (Level 3 KOs, Fig. 3c), K1190, K1810, K1892, and K1755 (Level K, Fig. 3d). These results implied that chronic noise changed phospholipid and galactose metabolism and were relevant to the oxidative status as well as the occurrence and development of EOAD pathology.

### Chronic noise exposure affects oxidative and inflammatory responses in the intestine of WT and APP/PS1 mice

**Fig. 4:**
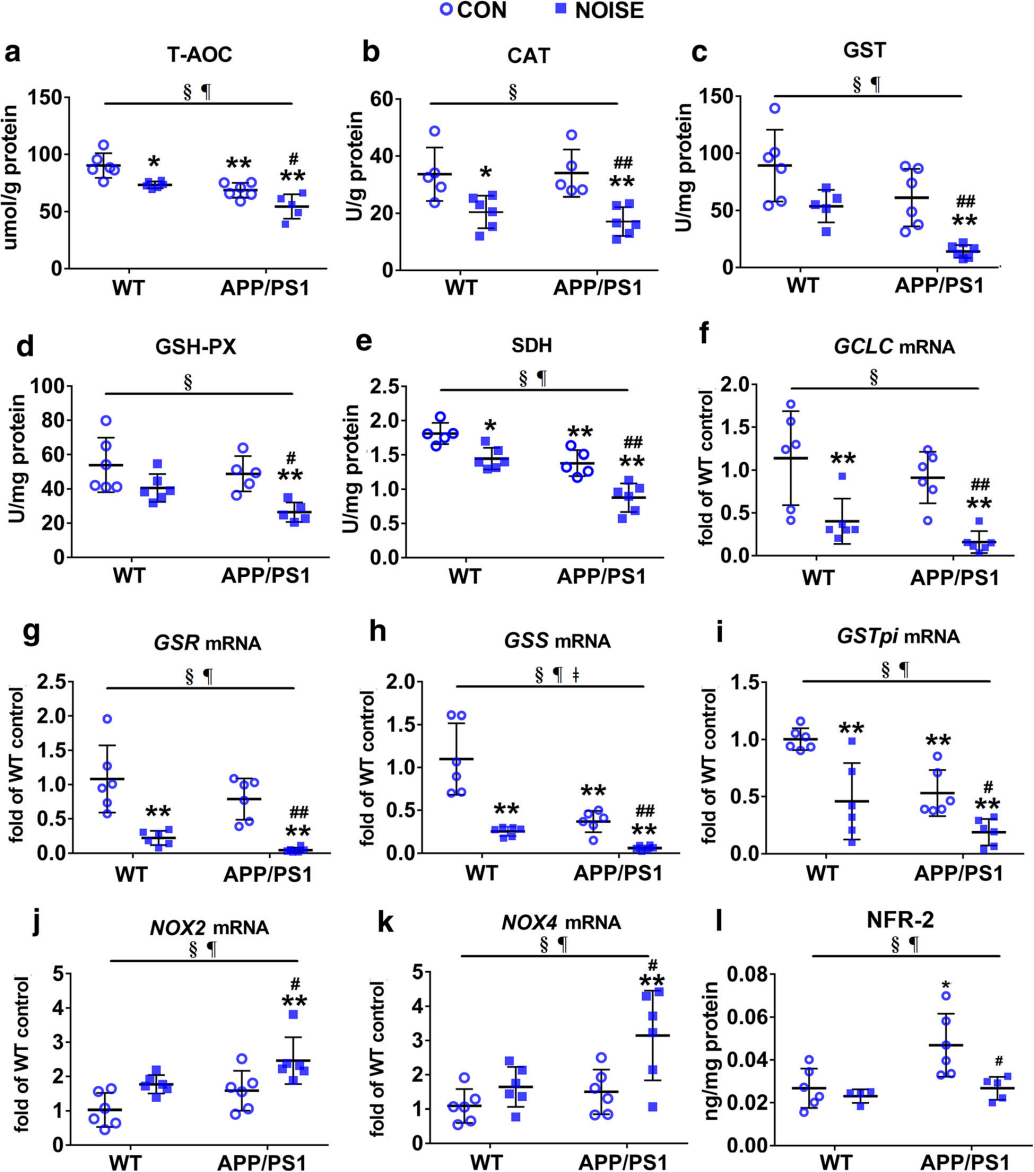
Effects of chronic noise exposure on the status of oxidative stress in the intestine. **a**–**e** Antioxidant capacities in cecal contents (*n* = 5–6). **f**–**k** mRNA expression levels of key enzymes in GSH/GST pathways and NOX2/4 in colon tissue (*n* = 6). **l** Enzyme-linked immunosorbent assay analysis of Nrf2 content in colon tissue (*n* = 5–6). Data are presented as the mean ± standard deviation. *P < 0.05, **P < 0.01 vs. WT CON group; ^#^*P* < 0.05, ^##^*P* < 0.01 vs. APP/PS1 CON group. §, exposure effect, *P* < 0.05; ¶, genotype effect, *P* < 0.05; ǂ, interaction effect, *P* < 0.05. CON, control groups; NOISE, noise-exposed groups; WT, wild-type mice; APP/PS1, APP/PS1 transgenic mice

To determine whether dysfunction of the gut–brain axis caused by intestinal flora dysbiosis after noise exposure was mediated by oxidative dysfunction, the gut homeostasis of the oxidation and anti-oxidation system was investigated. The activities of T-AOC, GST, CAT, and GSH-Px were significantly decreased in noise-exposed mice in both the WT and APP/PS1 groups, whereas the noise-induced downregulation of Nrf2 only occurred in APP/PS1 groups. APP/PS1 overexpression significantly decreased the activities of GST and T-AOC, with a trend towards decreasing activities of CAT and GSH-Px (Fig. 4a–d, l; ANOVA output: *T-AOC: exposure F(1,18) = 20.49*, *P* = 0.0002, *genotype F(1,18) = 34.54, P* < 0.0001, *interaction F(1,18) = 0.158*, *P* = 0.6955, *post hoc WT CON vs WT NOISE P* = 0.0093*, WT CON vs APP/PS1 CON P* = 0.0010*, WT CON vs APP/PS1 NOISE P* < 0.0001, *APP/PS1 CON vs APP/PS1 NOISE P* = 0.0175*; CAT: exposure F(1,18) = 24.53, P* = 0.0001, *genotype F(1,18) = 0.2323, P* = 0.6356, *interaction F(1,18) = 0.3593, P* = 0.5564*, post hoc WT CON vs WT NOISE P* = 0.0301, *WT CON vs APP/PS1 CON P* = 0.9998*, WT CON vs APP/PS1 NOISE P* = 0.0060, *APP/PS1 CON vs APP/PS1 NOISE P* = 0.0050; *GST: exposure F(1,18) = 10.47, P* = 0.0044, *genotype F(1,18) = 6.779, P* = 0.0175, *interaction F(1,18) = 0.5839, P* = 0.4542, *post hoc WT CON vs WT NOISE P* = 0.0632, *WT CON vs APP/PS1 CON P* = 0.1495, *WT CON vs APP/PS1 NOISE P* < 0.0001, *APP/PS1 CON vs APP/PS1 NOISE P* = 0.0070; *GSH-Px: exposure F(1,18) = 14.63, P* = 0.0012, *genotype F(1,18) = 4.286, P* = 0.0531, *interaction F(1,18) = 0.9393*, *P* = 0.3453, *post hoc WT CON vs WT NOISE P* = 0.1531, *WT CON vs APP/PS1 CON P* = 0.8805*, WT CON vs APP/PS1 NOISE P = 0.0013, APP/PS1 CON vs APP/PS1 NOISE P* = 0.0070*; Nrf2: exposure F(1,17) = 7.453, P* = 0.0143*, genotype F(1,17) = 7.501, P* = 0.0140*, interaction F(1,17) = 0.566, P* = 0.0762*, post hoc WT CON vs WT NOISE P* = 0.9358*, WT CON vs APP/PS1 CON P* = 0.0122*, WT CON vs APP/PS1 NOISE P* > 0.9999*, APP/PS1 CON vs APP/PS1 NOISE P* = 0.0169). Expression levels of *GCLC*, *GSR*, *GSS*, and *GSTpi* were affected by chronic noise exposure in both WT and APP/PS1 groups. APP/PS1 overexpression significantly reduced expression levels of *GSS* and *GSTpi* (Fig. 4f–i, ANOVA output: *GCLC*: exposure *F*(1,20) = 62.13, *P* < 0.0001, genotype *F*(1,20) = 3.738, *P* = 0.0675, interaction *F*(1,20) = 0.8248, *P* = 0.3746, *post hoc WT CON vs WT NOISE P* = 0.0072, *WT CON vs APP/PS1 CON P* = 0.6721, *WT CON vs APP/PS1 NOISE P* = 0.0005, *APP/PS1 CON vs APP/PS1 NOISE P* = 0.0060; *GSR*: exposure *F*(1,20) = 135.3, *P* < 0.0001, genotype *F*(1,20) = 8.739, *P* = 0.0078, interaction *F*(1,18) = 0.07271, *P* = 0.7902, *post hoc WT CON vs WT NOISE P* = 0.0003*, WT CON vs APP/PS1 CON P* = 0.3356*, WT CON vs APP/PS1 NOISE P* < 0.0001*, APP/PS1 CON vs APP/PS1 NOISE P* = 0.0015; *GSS*: exposure *F*(1,20) = 344.4, *P* < 0.0001, genotype *F*(1,20) = 210.3, *P* < 0.0001, interaction *F*(1,20) = 59.87, *P* < 0.0001, *post hoc WT CON vs WT NOISE P* < 0.0001*, WT CON vs APP/PS1 CON P* < 0.0001*, WT CON vs APP/PS1 NOISE P* < 0.0001*, APP/PS1 CON vs APP/PS1 NOISE P* = 0.1054; *GSTpi*: exposure *F*(1,20) = 28.14, *P* < 0.0001, genotype *F*(1,20) = 19.7, *P* = 0.0003, interaction *F*(1,20) = 1.404, *P* = 0.2499, *post hoc WT CON vs WT NOISE P = 0.0012, WT CON vs APP/PS1 CON P* = 0.0045*, WT CON vs APP/PS1 NOISE P* < 0.0001*, APP/PS1 CON vs APP/PS1 NOISE P* = 0.0463). Next to antioxidant capacity, the activity of the mitochondrial enzyme SDH was decreased markedly after noise exposure in both WT and APP/PS1 mice (Fig. 4e, exposure *F*(1,18) = 42.19, *P* < 0.0001, genotype *F*(1,18) = 31.86, *P* < 0.0001, interaction *F*(1,18) = 0.8051, *P* = 0.3814, *post hoc WT CON vs WT NOISE P* = 0.0168*, WT CON vs APP/PS1 CON P* = 0.0067*, WT CON vs APP/PS1 NOISE P* < 0.0001*, APP/PS1 CON vs APP/PS1 NOISE P* = 0.0011), while the expression of the *NOX2* and *NOX4*, the main resource of reactive oxygen species (ROS), was upregulated by noise exposure in APP/PS1 mice (Fig. 4j, k, *NOX2*: exposure *F*(1,20) = 8.443, *P* = 0.0087, genotype *F*(1,20) = 14.11, *P* = 0.0012, interaction *F*(1,20) = 0.09169, *P* = 0.7652; *NOX4*: exposure *F*(1,20) = 8.076, *P* = 0.0101, genotype *F*(1,20) = 10.65, *P* = 0.0039, interaction *F*(1,20) = 2.6, *P* = 0.1225, *post hoc WT CON vs WT NOISE P* = 0.6635*, WT CON vs APP/PS1 CON P* = 0.8205*, WT CON vs APP/PS1 NOISE P* = 0.0018*, APP/PS1 CON vs APP/PS1 NOISE P* = 0.0125), which may contribute to the progression of dysfunction of aerobic oxidative respiratory chain.

**Fig. 5:**
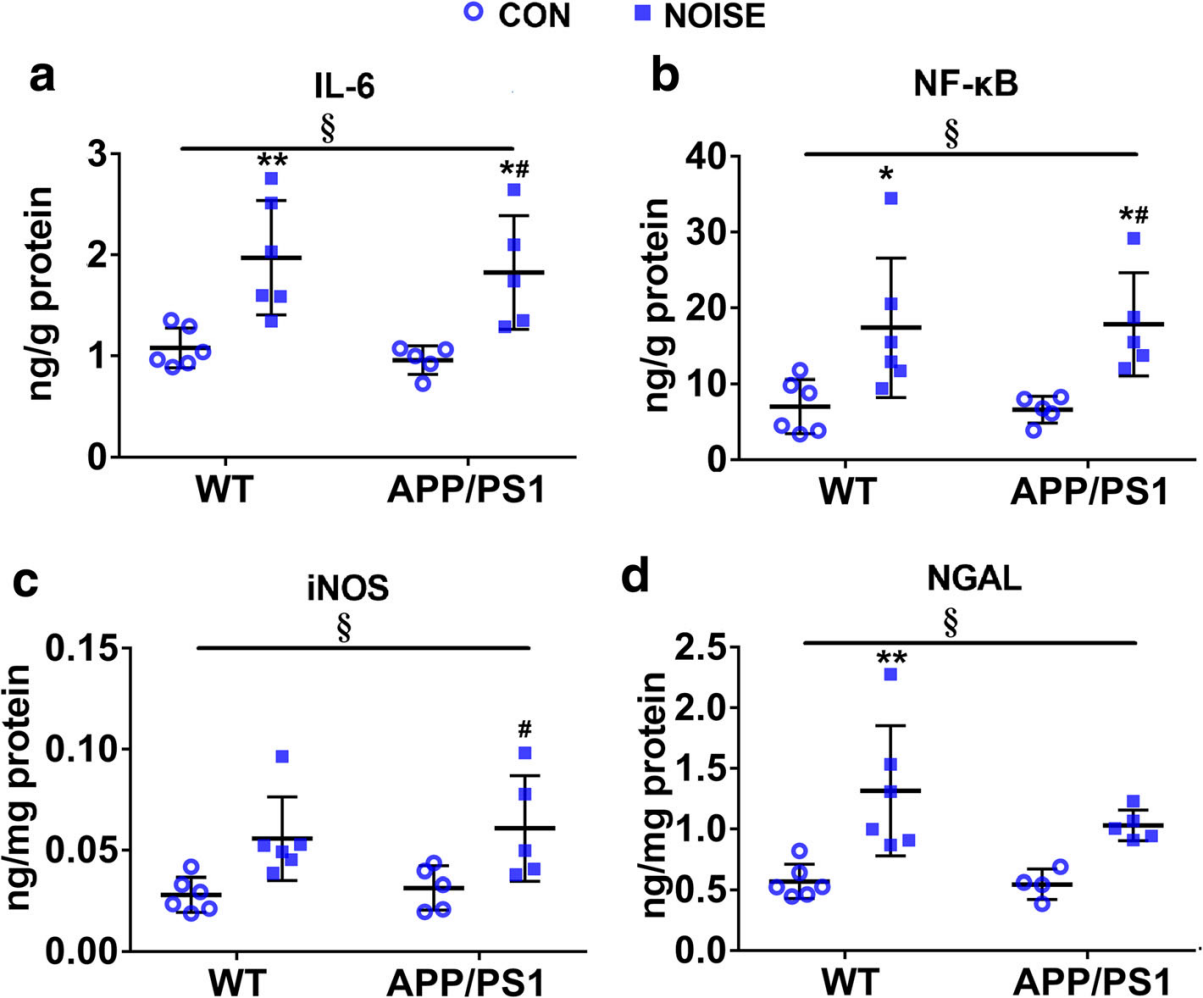
Intestinal abnormalities of host inflammatory responses following noise exposure. **a**–**d** Enzyme-linked immunosorbent assay analysis of inflammatory mediators (IL-6, NF-κB, iNOS, and NGAL) content in each group (*n* = 5–6). Data are presented as the mean ± standard deviation. *P < 0.05, **P < 0.01 vs. WT CON group; ^#^*P* < 0.05, ^##^*P* < 0.01 vs. APP/PS1 CON group. §, exposure effect, *P* < 0.05; ¶, genotype effect, *P* < 0.05; ǂ, interaction effect, *P* < 0.05. CON, control groups; NOISE, noise-exposed groups; WT, wild-type mice; APP/PS1, APP/PS1 transgenic mice

Oxidative stress and inflammatory responses reciprocally interact in a causal manner. We, thus, assessed the levels of gut inflammatory-associated mediators. The levels of IL-6, NF-κB, iNOS, and NGAL were significantly altered or showed a trend towards being altered by chronic noise and APP/PS1 overexpression (Fig. 5a–d, ANOVA output: IL-6: exposure *F*(1,18) = 24.27, *P* = 0.0001, genotype *F*(1,18) = 0.5591, *P* = 0.4643, interaction *F*(1,18) = 0.005064, *P* = 0.9441, *post hoc WT CON vs WT NOISE P* = 0.0080*, WT CON vs APP/PS1 CON P* = 0.9629*, WT CON vs APP/PS1 NOISE P* = 0.0386*, APP/PS1 CON vs APP/PS1 NOISE P* = 0.0195; NF-κB: exposure *F*(1,18) = 16.77, *P* = 0.0007, genotype *F*(1,18) = 1.862E-5, *P* = 0.9966, interaction *F*(1,18) = 0.02503, *P* = 0.8760, *post hoc WT CON vs WT NOISE P* = 0.0415*, WT CON vs APP/PS1 CON P* = 0.9995*, WT CON vs APP/PS1 NOISE P* = 0.0433*, APP/PS1 CON vs APP/PS1 NOISE P* = 0.0450; iNOS: exposure *F*(1,18) = 14.11, *P* = 0.0014, genotype *F*(1,18) = 0.3083, *P* = 0.5855, interaction *F*(1,18) = 0.0132, *P* = 0.9098, *post hoc WT CON vs WT NOISE P* = 0.0640*, WT CON vs APP/PS1 CON P* = 0.9892*, WT CON vs APP/PS1 NOISE P* = 0.0319*, APP/PS1 CON vs APP/PS1 NOISE P* = 0.0747; NGAL: exposure *F*(1,18) = 19.94, *P* = 0.0003, genotype *F*(1,18) = 1.268, *P* = 0.2758, interaction *F*(1,18) = 0.8862, *P* = 0.3597, *post hoc WT CON vs WT NOISE P* = 0.0034*, WT CON vs APP/PS1 CON P* = 0.9992*, WT CON vs APP/PS1 NOISE P* = 0.1069*, APP/PS1 CON vs APP/PS1 NOISE P* = 0.1314).

### Chronic noise-induced impairment of tight junctions in the intestine and hippocampus of WT and APP/PS1 mice

**Fig. 6:**
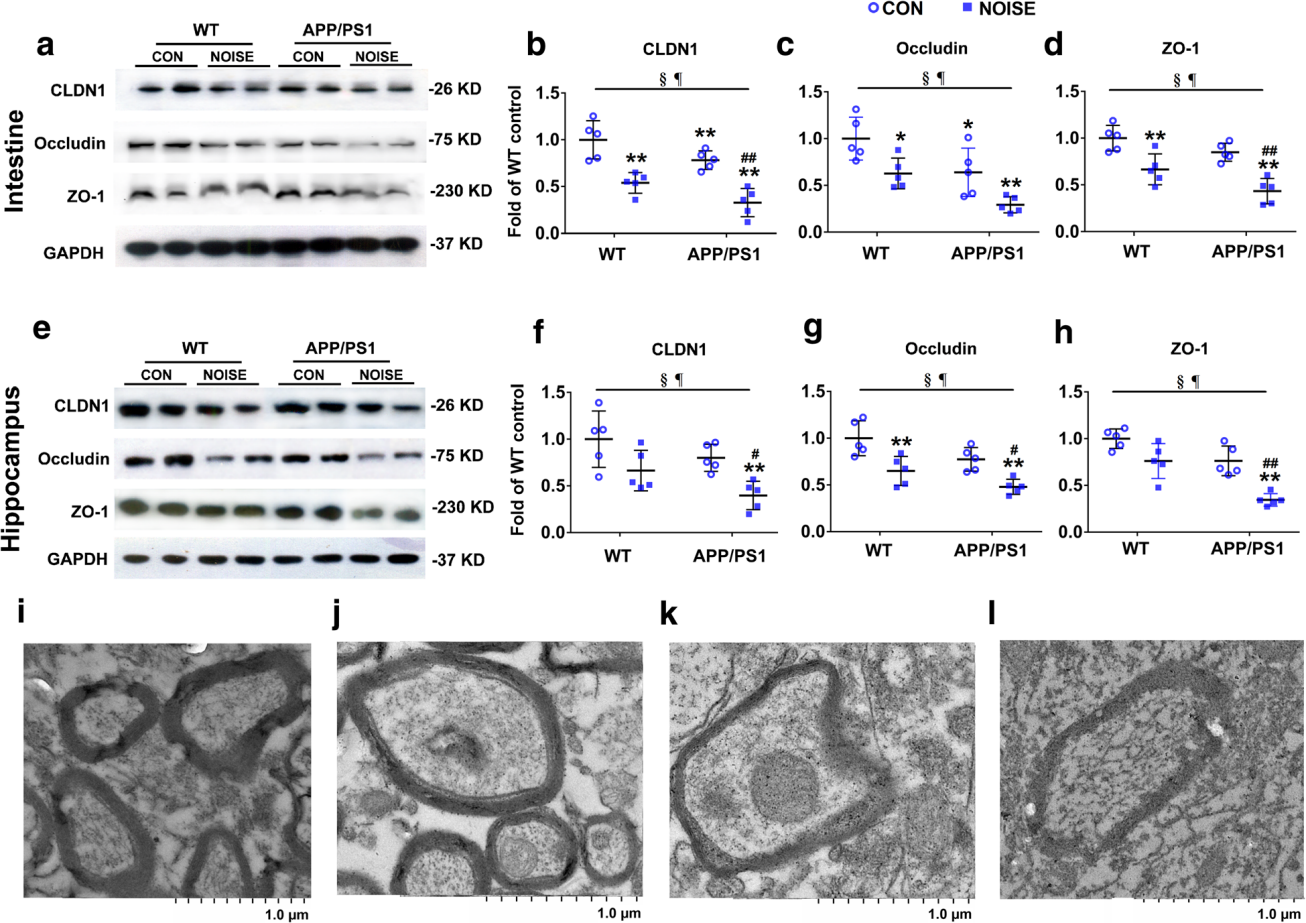
Chronic noise exposure diminishes tight junction protein expression in the intestine and hippocampus of WT and APP/PS1 mice. **a**–**d** Protein expression levels of the main tight junction components in intestine samples (*n* = 5). **e**–**h** Protein expression levels of main tight junction proteins in the hippocampus (*n* = 5). GAPDH was used as a loading control. Data are presented as the mean ± standard deviation. *P < 0.05, **P < 0.01 vs. WT CON group; ^#^*P* < 0.05, ^##^*P* < 0.01 vs. APP/PS1 CON group. §, exposure effect, *P* < 0.05; ¶, genotype effect, *P* < 0.05; ǂ, interaction effect, *P* < 0.05. CON, control groups; NOISE, noise-exposed groups; WT, wild-type mice; APP/PS1, APP/PS1 transgenic mice. **i**–**l** Morphology of the ultrastructural changes in tight junctions under transmission electron microscopy (magnification × 6000). **i** Wild-type, control groups. **j** APP/PS1 transgenic mice. **k** Wild-type, noise-exposed group. **l** APP/PS1 transgenic mice, noise-exposed group

To determine the status of intestinal and blood–brain barrier (BBB) permeability, we evaluated the expression levels of main tight junction proteins by using western blot analysis. Immunoblotting confirmed reduced intestinal expression of CLDN1, occludin, and ZO-1 in WT and APP/PS1 transgenic mice following noise exposure. APP/PS1 overexpression tended to decrease the levels of epithelial tight junction proteins (Fig. 6a–d, ANOVA output: CLDN1: exposure *F*(1,16) = 49.61, *P* < 0.0001, genotype *F*(1,16) = 10.91, *P* = 0.0045, interaction *F*(1,16) = 0.001969, *P* = 0.9652, *post hoc WT CON vs WT NOISE P* = 0.0007*, WT CON vs APP/PS1 CON P* = 0.1294*, WT CON vs APP/PS1 NOISE P* < 0.0001*, APP/PS1 CON vs APP/PS1 NOISE P* = 0.0008; occludin: exposure *F*(1,16) = 18.01, *P* = 0.0006, genotype *F*(1,16) = 16.83, *P* = 0.0008, interaction *F*(1,16) = 0.02282, *P* = 0.8818, *post hoc WT CON vs WT NOISE P* = 0.0378*, WT CON vs APP/PS1 CON P* = 0.0456*, WT CON vs APP/PS1 NOISE P* = 0.0002*, APP/PS1 CON vs APP/PS1 NOISE P* = 0.0563; ZO-1: exposure *F*(1,16) = 39.39, *P* < 0.0001, genotype *F*(1,16) = 10.29, *P* = 0.0055, interaction *F*(1,16) = 0.4828, *P* = 0.4971, *post hoc WT CON vs WT NOISE P* = 0.0065*, WT CON vs APP/PS1 CON P* = 0.3322*, WT CON vs APP/PS1 NOISE P* < 0.0001*, APP/PS1 CON vs APP/PS1 NOISE P* = 0.0009). Similar expression patterns of CLDN1, occludin, and ZO-1 were observed in the hippocampus of noise-exposed APP/PS1 mice. The expression of occludin in the hippocampus of noise-exposed WT mice was significantly reduced, while CLDN1 and ZO-1 expression only showed a trend to decrease without reaching significance (Fig. 6e–h, ANOVA output: CLDN1: exposure *F*(1,16) = 14.61, *P* = 0.0015, genotype *F*(1,16) = 5.796, *P* = 0.0285, interaction *F*(1,16) = 0.1203, *P* = 0.7332, *post hoc WT CON vs WT NOISE P* = 0.1001*, WT CON vs APP/PS1 CON P* = 0.4742*, WT CON vs APP/PS1 NOISE P* = 0.0020*, APP/PS1 CON vs APP/PS1 NOISE P* = 0.0394; occludin: exposure *F*(1,16) = 26.08, *P* = 0.0001, genotype *F*(1,16) = 9.861, *P* = 0.0063, interaction *F*(1,16) = 0.1906, *P* = 0.6682, *post hoc WT CON vs WT NOISE P* = 0.0066*, WT CON vs APP/PS1 CON P* = 0.0980*, WT CON vs APP/PS1 NOISE P* = 0.0002*, APP/PS1 CON vs APP/PS1 NOISE P* = 0.0227; ZO-1: exposure *F*(1,16) = 29.46, *P* < 0.0001, genotype *F*(1,16) = 29.05, *P* < 0.0001, interaction *F*(1,16) = 2.201, *P* = 0.1573, *post hoc WT CON vs WT NOISE P* = 0.0619*, WT CON vs APP/PS1 CON P* = 0.0650*, WT CON vs APP/PS1 NOISE P* < 0.0001*, APP/PS1 CON vs APP/PS1 NOISE P* = 0.0010). These data suggested that chronic noise-induced impairments in the epithelial integrity of the intestine and BBB open the gut–brain axis pathway of pathological substances, which may be synergistically aggravated by APP/PS1 overexpression.

Under transmission electron microscopy, the tight junctions between the cerebral microvascular endothelial cells in the control group were in a tight zipper shape, while the electron density of the junctions was high. However, a gap between WT and APP/PS1 mice was formed following noise exposure, while the density of electron-dense materials at the junction was decreased (Fig. 6i–l). These results suggest that noise exposure and APP/PS1 can damage the tight junction structure of vascular endothelial cells, thereby increasing BBB permeability.

### Abnormalities in neurotransmitters and inflammatory responses following noise exposure

**Fig. 7:**
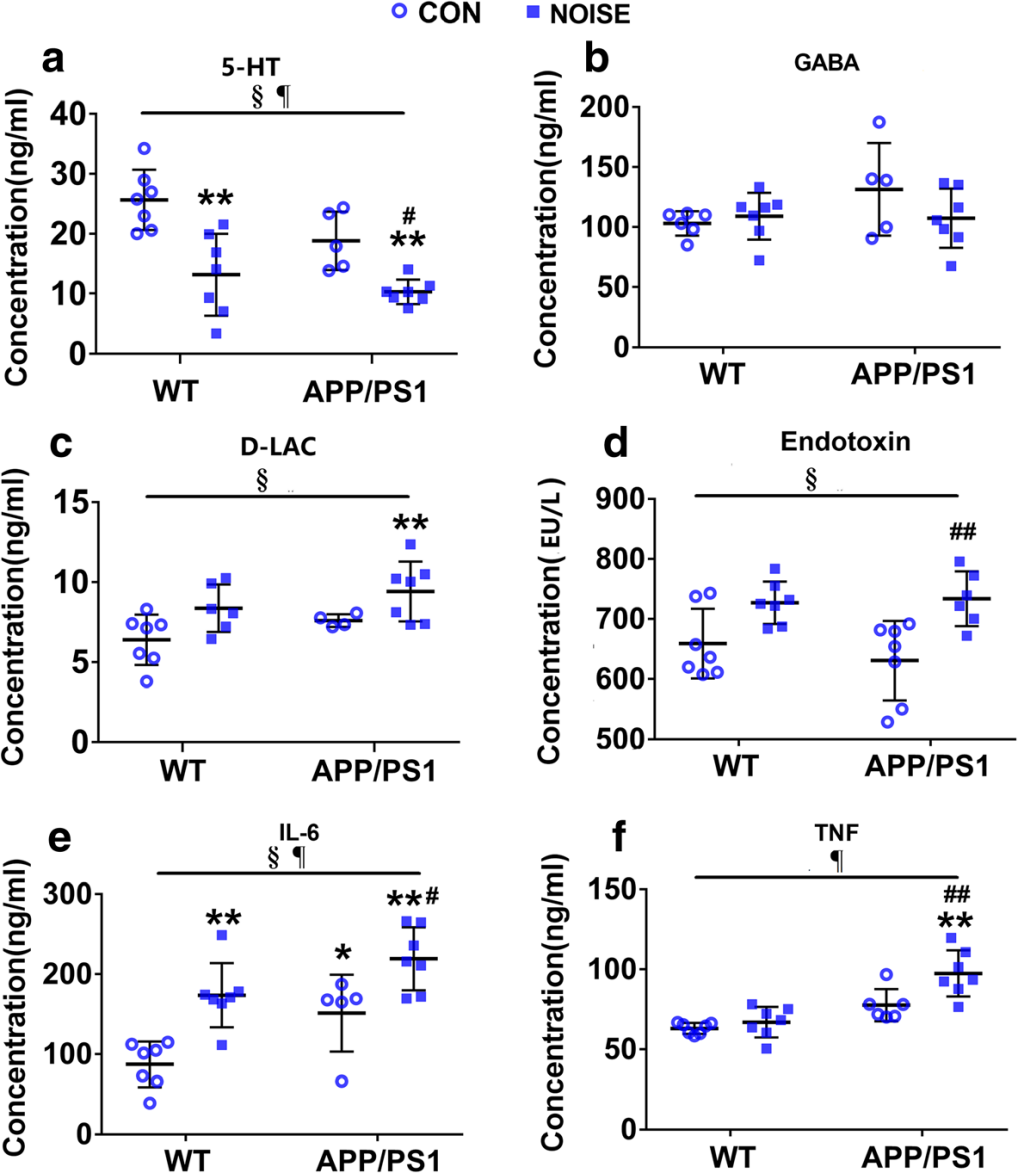
Circulatory abnormalities in neurotransmitters and inflammatory mediators following noise exposure. **a**–**f** Enzyme-linked immunosorbent assay analysis of the concentrations of neurotransmitters (5-HT and GABA) and inflammatory mediators (D-LAC, endotoxin, IL-6, and TNF) in each group (*n* = 5–7). Data are presented as the mean ± standard deviation. *P < 0.05, **P < 0.01 vs. WT CON group; ^#^*P* < 0.05, ^##^*P* < 0.01 vs. APP/PS1 CON group. §, exposure effect, *P* < 0.05; ¶, genotype effect, *P* < 0.05; ǂ, interaction effect, *P* < 0.05. CON, control groups; NOISE, noise-exposed groups; WT, wild-type mice; APP/PS1, APP/PS1 transgenic mice

To further explore the flora dysbiosis-triggered pathology response in the gut–brain axis, we examined serum levels of neurotransmitters (5-HT and GABA) and inflammatory mediators (D-LAC, endotoxin, IL-6, and TNF) using ELISAs. Noise exposure significantly decreased 5-HT concentrations in blood in both WT and APP/PS1 mice (Fig. 7a, ANOVA output: 5-HT: *exposure F*(1,23) = 28.04, *P* < 0.0001, *genotype F(1,23)* = 6.009, *P* = 0.0226*, interaction F(1,23)* = 1.009*, P* = 0.3261*, post hoc WT CON vs WT NOISE P* = 0.0006*, WT CON vs APP/PS1 CON P* = 0.1195*, WT CON vs APP/PS1 NOISE P* < 0.0001*, APP/PS1 CON vs APP/PS1 NOISE P* = 0.0381). The level of serum GABA showed a downward trend following noise exposure in APP/PS1 mice, but no significant difference was observed (Fig. 7b, ANOVA output: GABA: *exposure F(1,23)* = 0.8455*, P* = 0.3683*, genotype F(1,23) =* 1.844*, P* = 0.1889*, interaction F(1,23)* = 2.366*, P* = 0.1389, *post hoc WT CON vs WT NOISE P* = 0.9691*, WT CON vs APP/PS1 CON P = 0.2456, WT CON vs APP/PS1 NOISE P* = 0.9885*, APP/PS1 CON vs APP/PS1 NOISE P* = 0.3509). Additionally, significantly elevated serum levels of endotoxin, IL-6, and TNF were observed in noise-exposed APP/PS1 mice (Fig. 7d–f). The expression of IL-6 in noise-exposed WT mice was significantly increased, while no significant change was observed in endotoxin and TNF level (Fig. 7d–f, ANOVA output: endotoxin: *exposure F(1,23) =* 17.56*, P* = 0.0004*, genotype F(1,23) =* 0.285*, P* = 0.5986*, interaction F(1,23) =* 0.7259, *P* = 0.4030, *post hoc WT CON vs WT NOISE P* = 0.1034*, WT CON vs APP/PS1 CON P* = 0.7508*, WT CON vs APP/PS1 NOISE P* = 0.0806*, APP/PS1 CON vs APP/PS1 NOISE P* = 0.0098; IL-6: *exposure F(1,23) =* 25.14*, P* < 0.0001*, genotype F(1,23) =* 12.64*, P* = 0.0018*, interaction F(1,23) =* 0.3546, *P* = 0.5576, *post hoc WT CON vs WT NOISE P* = 0.0021*, WT CON vs APP/PS1 CON P* = 0.0466*, WT CON vs APP/PS1 NOISE P* < 0.0001*, APP/PS1 CON vs APP/PS1 NOISE P* = 0.0317; TNF: *exposure F(1,23) =* 9.067, *P* = 0.0062*, genotype F(1,23) =* 32.96*, P* < 0.0001*, interaction F(1,23) =* 4.145, *P* = 0.0534, *post hoc WT CON vs WT NOISE P* = 0.8945*, WT CON vs APP/PS1 CON P* = 0.0753*, WT CON vs APP/PS1 NOISE P* < 0.0001*, APP/PS1 CON vs APP/PS1 NOISE P* = 0.0097). In addition, noise exposure tended to increase serum D-LAC concentration in WT and APP/PS1 mice, but this did not reach significance (Fig. 7c, ANOVA output: *exposure F(1,20)* = 8.642*, P* = 0.0081, *genotype F(1,20) =* 3.043*, P* = 0.0964*, interaction F(1,20) =* 0.01405, *P* = 0.9068*, post hoc WT CON vs WT NOISE P* = 0.1308*, WT CON vs APP/PS1 CON P* = 0.6064*, WT CON vs APP/PS1 NOISE P* = 0.0076*, APP/PS1 CON vs APP/PS1 NOISE P* = 0.2653). These results suggested that chronic noise may affect gut microbiota-related neurochemistry and systemic inflammation, potentially contributing to the accumulation of AD-related pathology.

### Noise exposure induces AD-like neuropathology in the hippocampus of WT and APP/PS1 mice

**Fig. 8:**
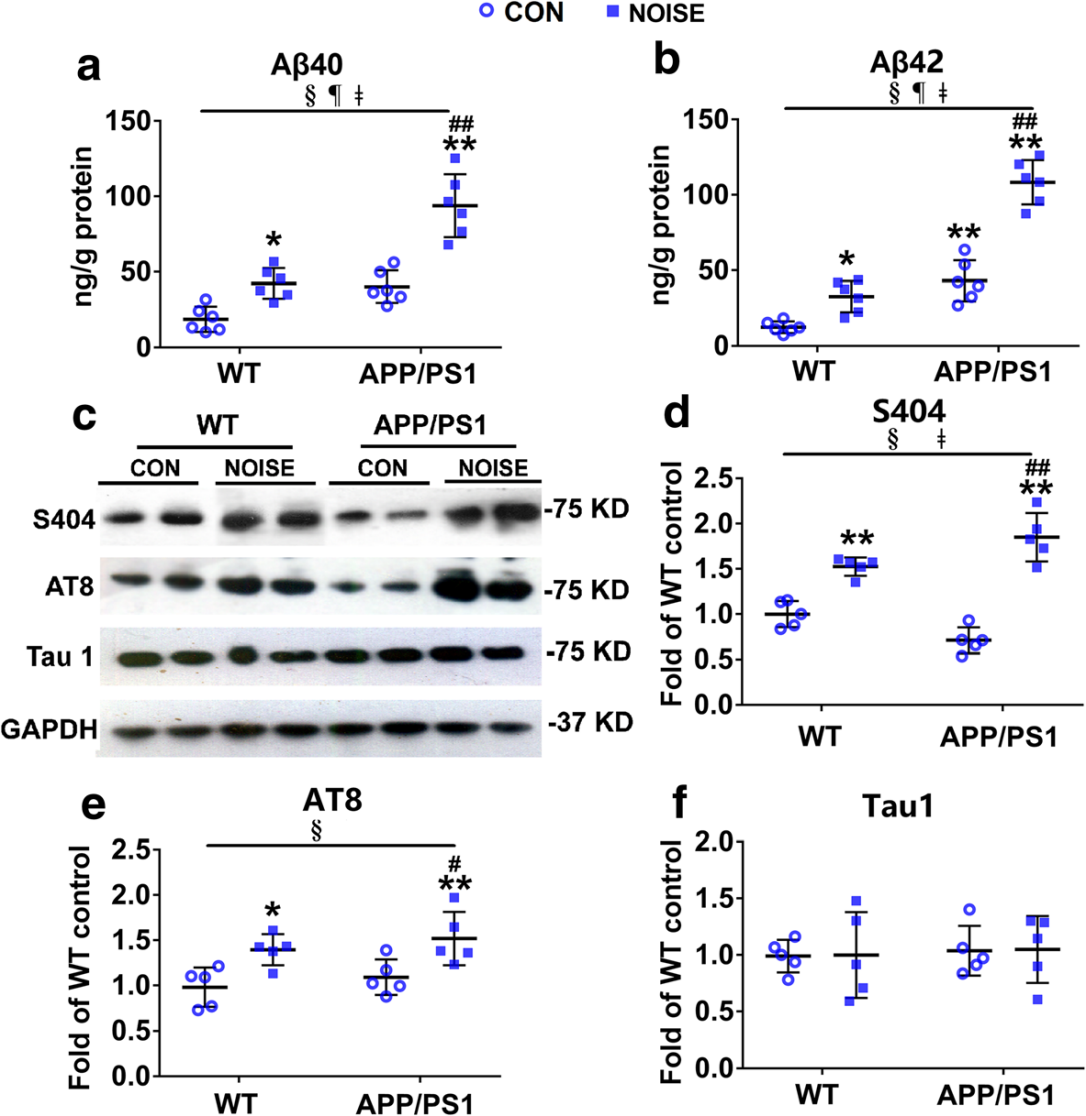
Chronic noise-induced Alzheimer’s disease-like neuropathological alterations in WT and APP/PS1 mice. **a**, **b** Enzyme-linked immunosorbent assay analysis of Aβ40 and Aβ42 content in each group (*n* = 6). **c**–**f** Western blot analysis (**c**) and quantification (**d**–**f**) of phosphorylated tau at AD-related sites in the hippocampus in each group (*n* = 5). GAPDH was used as a loading control. Data are presented as the percent change relative to control samples (*n* = 6). All data are presented as mean ± standard deviation. *P < 0.05, **P < 0.01 vs. WT CON group; ^#^*P* < 0.05, ^##^*P* < 0.01 vs. APP/PS1 CON group. §, exposure effect, *P* < 0.05; ¶, genotype effect, *P* < 0.05; ǂ, interaction effect, *P* < 0.05. CON, control groups; NOISE, noise-exposed groups; WT, wild-type mice; APP/PS1, APP/PS1 transgenic mice

To assess the effects of the CNS end of the gut–brain axis following chronic noise exposure, we examined levels of Aβ40 and Aβ42 using ELISA and relative expression of phosphorylated tau at AD-related sites by western blot analysis in hippocampal tissues. The hippocampal content of Aβ40 and Aβ42 was significantly elevated in the noise-exposed groups compared to those in the corresponding WT or APP/PS1 control groups (Fig. 8a and b, ANOVA output: *Aβ40: exposure F(1,20) = 49.57, P < 0.0001, genotype F(1,20) = 44.26, P < 0.0001, interaction F(1,20) = 7.396, P = 0.0132, post hoc WT CON vs WT NOISE P* = 0.0293*, WT CON vs APP/PS1 CON P* = 0.0518*, WT CON vs APP/PS1 NOISE P* < 0.0001*, APP/PS1 CON vs APP/PS1 NOISE P* < 0.0001; *Aβ42: exposure F(1,20) = 83.86, P < 0.0001, genotype F(1,20) = 130.1, P < 0.0001, interaction F(1,20) = 23.29, P = 0.0001, post hoc WT CON vs WT NOISE P* = 0.0288*, WT CON vs APP/PS1 CON P* = 0.0008*, WT CON vs APP/PS1 NOISE P* < 0.0001*, APP/PS1 CON vs APP/PS1 NOISE P* < 0.0001). Tau phosphorylation at Ser404, Ser202, and Thr205 sites was significantly higher in the noise-exposed groups than in the WT or APP/PS1 control groups (Fig. 8c–e, ANOVA output: Ser404: exposure *F*(1,16) = 112.5, *P* < 0.0001, genotype *F*(1,16) = 0.05214, *P* = 0.8223, interaction *F*(1,16) = 15.34, *P* = 0.0012, *post hoc WT CON vs WT NOISE P* = 0.0012*, WT CON vs APP/PS1 CON P* = 0.0804*, WT CON vs APP/PS1 NOISE P* < 0.0001*, APP/PS1 CON vs APP/PS1 NOISE P* < 0.0001; AT8: exposure *F*(1,16) = 17.36, *P* = 0.0007, genotype *F*(1,16) = 1.345, *P* = 0.2632, interaction *F*(1,16) = 0.004014, *P* = 0.9503, *post hoc WT CON vs WT NOISE P* = 0.0448*, WT CON vs APP/PS1 CON P* = 0.8625*, WT CON vs APP/PS1 NOISE P* = 0.0079*, APP/PS1 CON vs APP/PS1 NOISE P* = 0.0376). Neither exposure nor genotype affected protein expression of non-phosphorylated tau (Tau1) (Fig. 8f, ANOVA output: Tau1: exposure *F*(1,16) = 0.006968, *P* = 0.9345, genotype *F*(1,16) = 0.1539, *P* = 0.7001, interaction *F*(1,16) = 0.00006, *P* = 0.9941, *post hoc WT CON vs WT NOISE P* > 0.9999*, WT CON vs APP/PS1 CON P* = 0.9927*, WT CON vs APP/PS1 NOISE P* = 0.9864*, APP/PS1 CON vs APP/PS1 NOISE P* > 0.9999). These data suggested that chronic noise exposure and APP/PS1 genotype aggravated AD-related pathological alterations in a synergistic manner.

## Discussion

In this study, we confirmed that chronic noise can cause a series of microbiome–gut–brain axis events in the EOAD transgenic mice model, including abnormalities in the composition of gut microbial community and oxidative-inflammatory state, disrupted tight junction function in the intestine and BBB, and systemic inflammatory responses associated with amyloid pathology and tau hyperphosphorylation. Moreover, we found evidence that combined exposure to environmental noise stress and APP/PS1 overexpression may synergistically result in a systemic oxidative-inflammatory status driven by shifts in microbial load and metabolism, which may add a new dimension to certain biological markers of AD pathology to facilitate the onset or progression of EOAD.

### Noise exposure contributes to the gut microbiota dysbiosis and possibly alters homeostasis of oxidation-inflammation

The composition of intestinal flora is easily influenced by environmental factors [20], which is a susceptible event that drives subsequent gut–brain axis effects. Our results demonstrated that environmental noise exposure not only aggravated abnormalities in the gut microbiota composition but also significantly decreased the abundance of *Rikenellaceae* and *Ruminococcaceae* families as well as that of *Anaerovorax*, *Lachnospira*, and *Odoribacter* genus in microbial communities, particularly in the APP/PS1 overexpression groups. Moreover, chronic noise exposure increased the abundance of *Staphylococcus*, a conditional pathogen that has previously been reported to be associated with elevated endotoxemia [21], which is known to increase inflammatory cytokines, cause endothelial dysfunction in humans, and be associated with metabolic syndrome [22]. The abundance of *Rikenellaceae* was negatively correlated with metabolism and cardiovascular diseases [23]. Notably, other bacteria with significant changes in abundance belong to butyrate-producing groups [24, 25]. Interestingly, a typical feature of intestinal microflora imbalance is a decrease in the abundance of common butyrate-producing bacteria and an increase in the number of conditional pathogens [26]. Butyrate is an energy source for epithelial cells and can protect cells from oxidative stress; it maintains redox homeostasis and mitochondrial energy metabolism through a regulation of the GSK-3β/Nrf2 pathway [27]. A human study further demonstrated that colonic administration of butyrate primarily resulted in an increased transcriptional regulation of the pathways representing fatty acid oxidation, electron transport chain, and oxidative stress [28]. Moreover, butyrate intervention influences inflammation [27], epithelial integrity, and apoptosis [29]. The present findings suggest that intestinal flora dysbiosis-related oxidative damage may play a key role in the subsequent gut–brain axis effects induced by noise exposure, which may have important etiological implications.

We observe that the dysfunction in KEGG pathways related to the metabolism of phospholipid and galactose in functional predictions via Tax4Fun is consistent with the findings of a previous study showing that metabolic mechanisms of stress-related cognitive impairments in APP/PS1 mice were partly related to sphingolipid metabolism [30]. Abnormal phospholipid metabolism is closely related to oxidative stress and inflammatory responses and plays a significant role in the occurrence and development of cardiovascular and cerebrovascular diseases [31]. Accumulation of D-galactose triggers ROS generation and leads to oxidative stress and inflammation, which is widely used to mimic aging in animal models [32]. A comprehensive analysis of the aforementioned results of flora composition and functional prediction highlight pathways corresponding to oxidative stress and the development of cell senescence, although the Tax4Fun prediction analysis has its limitations in accuracy and the underlying mechanisms need further elucidation. If true, this hypothesis would imply that systemic inflammatory responses and subsequent gut–brain axis disruption by environmental noise could be mediated by microbiota dysbiosis involving oxidation-inflammation pathways.

Recent researches have demonstrated that gut microbiota shift toward proinflammatory bacteria in APP/PS1 transgenic mice [33], and influence mitochondrial function by regulating ROS production [34, 35], suggesting that the intestinal microbiota-regulated homeostasis of oxidation-inflammation plays key roles in EOAD pathogenesis [36]. The cellular mechanism of ROS overproduction is closely related to mitochondrial dysfunction, which is promoted by defective electron transport chain enzymes (i.e., SDH and NOX) and inactivation of antioxidant mitochondrial enzymes (i.e., GSH, GST, and CAT) [37, 38]. Moreover, Nrf2-related signaling is a key factor in oxidative stress, which regulates the enzymes involved in antioxidative stress responses by interacting with antioxidant response elements and underlies cellular repair mechanisms in inflammatory states [39]. There exists a bidirectional relationship between oxidative stress and inflammation, oxidative impairment can be induced by inflammatory responses, and inflammation can be triggered or aggravated by ROS through activation of NF-κB, which controls the expression of genes involved in inflammatory responses [40]. Hence, altered microbial populations may dysregulate redox balance and consequently cause inflammatory responses. Our previous data demonstrated systemic inflammatory responses [16, 19] and reciprocal activation of proinflammatory cytokines and astrocytes following noise exposure, which could cause a positive feedback loop, resulting in continuous accumulation of low-level neuropathology [9]. In this study, we confirmed that environmental noise or APP/PS1 overexpression increased intestinal oxidative stress via dysregulation of Nox2/4 and antioxidant-related enzymes. In either scenario, noise-induced abnormalities, including oxidative stress, systemic inflammation, and a trend for increases in serum D-LAC, were consistent with the results of flora composition and functional prediction, again suggesting that the dysbiosis of intestinal flora initiates gut–brain axis effects caused by noise exposure. Importantly, a recent study demonstrated that Nrf2 deficiency impairs barrier integrity by disrupting the energy-dependent tight junction [41]. Thus, it is reasonable to postulate that noise-inducedNrf2-related antioxidative dysfunction may also contribute to tight junction pathology in the gut–brain axis.

### Noise exposure facilitates AD-like pathologies through disruption of the gut–brain axis in EOAD model mice

The present study revealed that both chronic noise exposure and APP/PS1 overexpression caused dysregulation of the expression of tight junction proteins, including claudin 1, occludin, and ZO-1, in the intestine and hippocampus, demonstrating widespread deficiency in intestinal barrier function and BBB integrity. The epithelial tight junction transmembrane proteins (claudins and occludin) and cytoplasmic membrane proteins (ZO-1 and ZO-2) maintain the permeability function of the BBB and intestinal barrier, which can be downregulated by oxidative stress on inhibition of CaMKKβ-AMPK signaling [42]. Moreover, oxidative stress can directly affect permeability and influence homeostasis of the intestinal epithelial barrier, thus increasing the risk of intestinal flora reaching the circulatory system and CNS through the gut–brain axis [43]. Thus, these results at least partly explain the elevated serum levels of xenobiotic molecules and inflammatory mediators, which play a major role in the low-grade systemic inflammation of the gut–brain axis and neurochemical dysregulation underscoring neurodegeneration.

Gut microbiota dysbiosis and its induction of oxidative and inflammatory imbalance triggers a cascade response in the gut–brain axis due to BBB disruption and aggravates AD-like cerebral pathology via immune, metabolite-mediated, and oxidative signaling pathways [44]. A recent study has suggested that loss of cortical tight junction proteins occurs in association with the accumulation of AD-related insoluble proteins, particularly Aβ [45]. Moreover, it is noteworthy that the loss of tight junction proteins was associated with a loss of synaptic markers [45], which play key roles in cognitive decline in AD. Since AD is characterized by the progressive accumulation of insoluble proteins and cognitive deterioration, it is reasonable to postulate that tight junction dysfunction in the gut–brain axis may contribute to the onset or/and progression of AD-like neuropathology. Our results indicate that the presence of dysbiosis and oxidative stress, causing the breakdown of the intestinal and BBB permeability and systemic spread of inflammatory condition, accelerated Aβ production and tau hyperphosphorylation in a synergistic manner with APP/PS1 overexpression. The downregulation of serum 5-HT and GABA in noise-exposed mice closely associated with gut microbiota disruption and enhanced hippocampal Aβ synthesis provide further evidence for the microbiome–gut–brain axis underlying the mechanism of AD development. Additionally, we demonstrated that 30 days of exposure to environmental noise was sufficient to facilitate the occurrence and development of AD-like pathologies in 3-month-old APP/PS1 Tg mice, which have an accelerated EOAD phenotype characterized by elevated Aβ deposits at an age as young as 13–16 weeks old [17].

The mutations in PSEN1, PSEN2, or APP genes can only explain the pathogenesis of a small number of cases, and their precise contribution is not fully clarified. Environmental stress increased glucocorticoid levels, AD-like neuropathology, and cognitive impairments in APP/PS1 mice, implying that EOAD model mice are more vulnerable to environmental stress than WT mice [13]. This line of thinking also raises the possibility that the synergy between environmental noise and APP/PS1 overexpression may underlie the pathogenesis of EOAD. To our knowledge, there is scant epidemiological evidence for the relationship between environmental stressor exposure and EOAD development. Experimental data based on the APP/PS1 EOAD model, including data from this study, may provide important clues in this regard.

### Limitations of study

Although we have initially constructed a chain through the analysis methods, such as Tax4Fun function prediction, combined with evidence from previous studies, the causal relationship between some results presented herein lacks direct experimental evidence support. Further studies should be conducted on the butyric acid producing bacteria to confirm whether the altered composition of gut microbiota and deduced phospholipid and galactose metabolism are indeed causally linked to oxidative responses, decreased expressions of intestinal and hippocampal tight junction proteins, and the increased hippocampal Aβ40 and Aβ42.

## Conclusions

Taken together, exposure to long-term environmental noise, likely via the associated alterations in microbiome–gut–brain axis status, can exacerbate amyloid and tau pathology in early-aged EOAD model mice. Noise exposure upregulates oxidative stress and systemic low-grade inflammation, which may underlie epithelial barrier deficits in the intestine and brain, and may thus be an environmental risk factor for EOAD in vulnerable individuals. These findings expand our understanding of the etiological signaling pathways involved in EOAD pathology following environmental hazard stress. Further, they suggest that low-grade oxidative stress and inflammatory responses in the gut–brain axis are potential biochemical and pathological markers in the presymptomatic stage of EOAD. Early identification may thus help protect against the detrimental effects of chronic noise exposure.

## Data Availability

Raw data is available from the corresponding authors upon reasonable request.

## Abbreviations

AD: Alzheimer’s disease
EOAD: Early-onset Alzheimer’s disease
Aβ: Amyloid-β
PSEN1: Presenilin 1
PSEN2: Presenilin 2
APP: Amyloid precursor protein
APP/PS1: B6C3-Tg (APPswe, PSEN1dE9)/NJU
WT: Wild-type (C57BL/6Nju)
SPL: Sound pressure level
OTU: Operational taxonomic unit
LEfSe: Linear discriminant analysis LAD coupled with effect size measurements
T-AOC: Total antioxidant capacity
CAT: Catalase
GST: Glutathione S-transferase
GSH-Px: Glutathione peroxidase
Ct: Threshold cycle
ELISA: Enzyme-linked immunosorbent assay
GADPH: Glyceraldehyde phosphate dehydrogenase
KO: KEGG ortholog
BBB: Blood–brain barrier
SAMP8: Senescence accelerated prone
NF-κB: Nuclear factor-kappa B
